# Clinical Outcomes and Hospital Resource Utilization Among Patients with Sepsis and COVID-19 Infection: Insights from the United States National Inpatient Sample Analysis, 2020–2022

**DOI:** 10.3390/idr18050118

**Published:** 2026-10-10

**Authors:** Antony Arumairaj, Dili Dhanani, Fatema Ali Asgar Tashrifwala, Anuradha Shunmugam Veluswamy, Aniroodh Venugopalan, Radhika Annam, Poojaben Dhorajiya, Vimala Sravanthi Vajjala

**Affiliations:** 1 Department of Medicine, Woodhull Medical Center, Brooklyn, NY 11206, USA; 2 Department of Anesthesiology, University of Kansas Hospital, Kansas City, KS 66160, USA; ddhanani@kumc.edu; 3 Department of Medicine, Stamford Hospital, Stamford, CT 06902, USA; fatema.tashrifwala12@gmail.com; 4 Department of Medicine, Southcoast Health, New Bedford, MA 02740, USA; swamyfamy@gmail.com; 5 Department of Medicine, Rajiv Gandhi University of Health Sciences, Bangalore 560041, India; aniroodh.doc@gmail.com; 6 Department of Medicine, Hospital Sisters Health System Medical Group, Springfield, IL 62711, USA; radhika.annam87@gmail.com; 7 Department of Pathology and Laboratory Medicine, University of Kansas Medical Center, Kansas City, KS 66160, USA; pdhorajiya@kumc.edu; 8 Department of Neurology, Banner University Medical Center, University of Arizona, Phoenix, AZ 85006, USA; vajjalavs@gmail.com

**Keywords:** COVID-19 pandemic, sepsis, in-hospital mortality, acute kidney injury (AKI), renal replacement therapy (RRT), acute respiratory distress syndrome (ARDS), extracorporeal membrane oxygenation (ECMO), National Inpatient Sample (NIS) database

## Abstract

Background/Objectives: Clinical studies have shown that patients who had sepsis with COVID-19 infection had worse clinical outcomes; however, there is a paucity of data on the association between concurrent COVID-19 infection and outcomes among patients with sepsis. Hence, we conducted a retrospective cohort study using the National Inpatient Sample (NIS) database from 2020 through 2022 to evaluate the association between concurrent COVID-19 infection and outcomes among patients with sepsis. Methods: Adult hospitalizations with sepsis were identified using ICD-10-CM codes; hospitalizations with septic shock were excluded, and the remaining cohort was stratified by concurrent COVID-19 infection status. Survey-weighted multivariate regression analysis was performed. Results: Patients with sepsis with concurrent COVID-19 infection had worse outcomes with higher mortality rate (15.6% vs. 5.4%; aOR 3.70; 95% CI 3.62–3.79), higher need for invasive ventilation (13.1% vs. 6.5%) and NIV (non-invasive ventilation) (10.2% vs. 4.9%), higher incidence of acute kidney injury (37.6% vs. 36.7%), acute respiratory failure (57.5% vs. 20.3%), disseminated intravascular coagulation (DIC) (0.4% vs. 0.3%), cardiac arrest (3.1% vs. 1.5%), acute respiratory distress syndrome (ARDS) (7.9% vs. 0.4%), higher need for renal replacement therapy (4.9% vs. 4.5%), tracheostomy (1.6% vs. 0.6%), extracorporeal membrane oxygenation (ECMO) (0.2% vs. 0.04%), longer length of hospital stay (10.3 days vs. 7.6 days), and higher total charges ($127,632 vs. $93,735, respectively). Conclusions: The results demonstrate that COVID-19 infection was independently associated with worse clinical outcomes among those hospitalized with sepsis. These results underscore the need for early recognition, aggressive treatment, and targeted therapy in this high-risk clinical group.

## 1. Introduction

The COVID-19 pandemic has resulted in more than seven million deaths worldwide and nearly 1.2 million deaths in the United States, making it one of the most significant public health crises in recent history [1]. Sepsis is a manifestation of severe COVID-19 infection and has contributed to worse clinical outcomes resulting in increased morbidity and mortality. An analysis of U.S. death certificate data demonstrated that sepsis was reported in 9.3% of COVID-19 associated deaths, highlighting the lethal role of sepsis in COVID-19-associated mortality [2].

Sepsis continues to be a leading cause of morbidity, mortality, and healthcare expenditure worldwide [3]. COVID-19 associated sepsis is characterized by cytokine storm, which is a dysregulated and exaggerated host immune response to SARS-CoV-2 infection. This hyperinflammatory state is characterized by elevated levels of pro-inflammatory cytokines, including interleukin IL-6, IL-1β, IL-8, and tumor necrosis factor (TNF)-α, causing widespread immune hyperactivity and systemic inflammation, endothelial injury, complement activation, microvascular thrombosis and disseminated intravascular coagulation (DIC), which collectively result in tissue hypoperfusion and multiorgan failure [4,5,6,7].

The clinical presentation and management of COVID-19-associated sepsis differ significantly from those of conventional bacterial sepsis, as SARS-CoV-2 induces profound immune dysregulation and increases susceptibility to secondary bacterial infections. Patients with COVID-19-associated sepsis often develop severe complications of hypoxemic respiratory failure, acute respiratory distress syndrome (ARDS), shock, and multiorgan failure, frequently requiring advanced life support and prolonged hospitalization [8]. These distinct clinical features indicate that concurrent COVID-19 infection may substantially increase the severity of sepsis leading to higher healthcare resource utilization, and increased mortality among hospitalized patients with sepsis.

Despite growing recognition of COVID-19-associated sepsis in patients with severe COVID-19 infection, its nationwide association with clinical outcomes and healthcare resource utilization across the United States remains incompletely characterized. Most clinical studies evaluating clinical outcomes associated with COVID-19-associated sepsis have been limited to single center studies and local and regional healthcare systems, thereby restricting the generalizability of their findings and conclusions [9]. In addition, the emergence of new SARS-CoV-2 variants, enhanced vaccination coverage, and advances in supportive treatment have significantly altered the epidemiology and outcomes of COVID-19, highlighting the need for a more comprehensive nationwide analysis.

The National Inpatient Sample (NIS), the largest publicly available all-payer inpatient database in the United States, provides a unique opportunity to evaluate the association between concurrent COVID-19 infection and clinical outcomes and healthcare resource utilization among patients hospitalized with sepsis nationwide. Therefore, we conducted a retrospective cohort study using the NIS database from 2020 through 2022 to compare clinical outcomes and hospital resource utilization between patients hospitalized with sepsis and concurrent COVID-19 infection and those hospitalized with sepsis without COVID-19 infection, and to determine the trends in clinical outcomes from the early pandemic in 2020 to 2022.

## 2. Methods

### 2.1. Study Design and Data Source

We conducted a retrospective cohort study using data from the National Inpatient Sample (NIS) database from January 2020 through December 2022. The NIS is the largest inpatient database in the United States and employs a survey design that collects discharge information from non-federal, non-rehabilitation, acute care, and short-term hospitals. The database is derived from hospital billing data submitted to statewide data organizations across the United States and covers more than 97% of the U.S. population. Institutional Review Board approval was not required because the NIS is a publicly available de-identified database, and the study complied with the NIS data use agreement.

### 2.2. Study Population

Adult hospitalizations (≥18 years) with sepsis were identified using the International Classification of Diseases, Tenth Revision, Clinical Modification (ICD-10-CM) diagnosis codes A40 (streptococcal sepsis) and A41 (other sepsis, including staphylococcal, anaerobic, gram-negative, other specified, and unspecified organisms), together with code R65.20 for severe sepsis without septic shock recorded in any diagnosis position. Hospitalizations with septic shock (ICD-10-CM code R65.21) were excluded. Concurrent COVID-19 infection was identified using ICD-10-CM diagnosis code U07.1.

The study population was stratified into two groups based on the presence or absence of concurrent COVID-19 infection during the index hospitalization.

### 2.3. Variables and Outcomes

The primary outcome of interest was in-hospital mortality. Secondary outcomes included invasive mechanical ventilation, non-invasive ventilation (NIV), acute kidney injury (AKI), renal replacement therapy (RRT), cardiac arrest, acute respiratory distress syndrome (ARDS), extracorporeal membrane oxygenation (ECMO), acute respiratory failure, acute liver failure, disseminated intravascular coagulation (DIC), tracheostomy, length of hospital stay, total hospital charges, and discharge disposition. Variables included age, sex, race, median household income quartile by ZIP code, primary insurance status, Charlson Comorbidity Index (CCI), categorized as 0, 1, 2, or ≥3, hospital region, hospital location and teaching status, and hospital bed size. We also assessed annual trends in hospitalization distribution and in-hospital mortality from 2020 through 2022.

### 2.4. Statistical Analysis

Patient information and study data were analyzed using Stata/BE version 17.0 (StataCorp LLC, College Station, TX, USA). The NIS employs a complex survey design that incorporates stratification, clustering, and discharge-level sampling weights to generate nationally representative estimates. Accordingly, all analyses were performed using NIS-recommended survey methodology. Continuous variables were reported as weighted means and compared using survey-weighted linear regression with adjusted Wald tests. Categorical variables were presented as weighted percentages and compared using the Rao–Scott corrected chi-square test. Survey-weighted multivariable logistic regression analyses were performed to estimate adjusted odds ratios (aORs) with 95% confidence intervals (CIs) for binary outcomes. Survey-weighted multivariable linear regression analyses were used to evaluate adjusted mean differences for length of hospital stay and total hospital charges. Length of stay and total hospital charges were right-skewed; therefore, distributional diagnostics were assessed and sensitivity analyses were performed using survey-weighted generalized linear models with a gamma distribution and log link. Survey-weighted linear regression was retained as the primary approach because it provides directly interpretable adjusted absolute mean differences in resource utilization. All multivariable models were adjusted for age, sex, race, insurance status, median household income quartile, Charlson Comorbidity Index, hospital region, hospital location and teaching status, hospital bed size and calendar year. Statistical significance was defined as a two-sided *p*-value < 0.05.

## 3. Results

**Figure 1:**
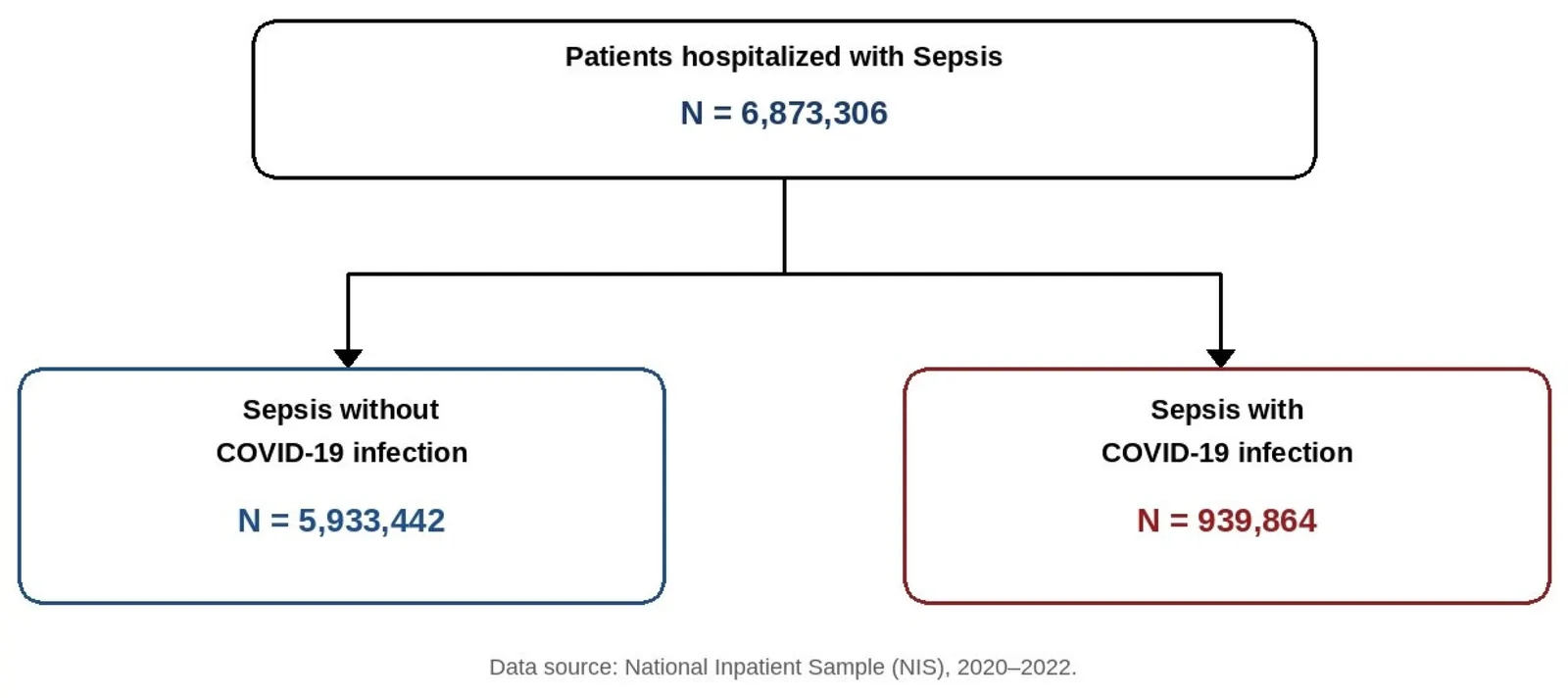
Flowchart of patient distribution in this study.

A total of 5,934,565 weighted adult COVID-19 hospitalizations and a total of 6,873,306 weighted adult hospitalizations with sepsis were identified from the 2020–2022 National Inpatient Sample database. The 6,873,306 patients with diagnoses of sepsis were stratified based on the presence or absence of COVID-19 infection; 939,864 had concomitant COVID-19 infection, whereas 5,933,442 did not have concomitant COVID-19 infection [Figure 1].

Baseline demographic and hospital characteristics are summarized in Table 1. In the overall adult sepsis cohort, the mean age was 63.8 (17.6) years, 52.3% were male, and the largest racial group was Caucasian (65.6%), followed by African American patients (14.8%) and Hispanic patients (12.8%). The largest proportion of patients belonged to the lowest household income quartile (31.1%), Medicare was the most common primary payer (58.9%), and most patients had a Charlson Comorbidity Index (CCI) of ≥3 (43.7%). Most hospitalizations occurred in the South (41.6%), at urban teaching hospitals (71.0%), and in large hospitals (45.6%). In the overall cohort, A40 streptococcal sepsis accounted for 2.8% of hospitalizations, while A41 other sepsis accounted for 97.1% (Supplementary Table S1).

Patients with sepsis and concomitant COVID-19 infection were of similar age compared with those without COVID-19 infection (63.8 [17.2] vs. 63.8 [17.7] years, *p* = 0.82). A40 streptococcal sepsis was less common in the COVID-19 sepsis cohort than in the non-COVID-19 sepsis cohort (1.0% vs. 3.1%, *p* < 0.001), whereas A41 other sepsis was more common in the COVID-19 sepsis cohort (98.9% vs. 96.8%, *p* < 0.001); stratified A40/A41 subgroup analyses are presented in Supplementary Table S2. The sex distribution was higher in males, accounting for the majority of hospitalizations in both groups (55.6% vs. 51.8%) (*p* < 0.001). Caucasians constituted the largest racial group in both cohorts (55.8% vs. 67.2%), followed by African American patients (17.6% vs. 14.3%) (*p* < 0.001). The largest proportion of patients belonged to the lowest household income quartile (31.7% vs. 31.0%), followed by the second income quartile (26.3% vs. 26.2%) (*p* < 0.001). Medicare was the most common primary payer in both groups (55.2% vs. 59.4%), followed by private insurance (26.0% vs. 19.5%) (*p* < 0.001). Most patients had a Charlson Comorbidity Index (CCI) of ≥3 (35.6% vs. 44.9%), followed by a CCI of 1 (24.1% vs. 18.9%) (*p* < 0.001). Most hospitalizations occurred in the Southern region (43.2% vs. 41.4%), followed by the West (22.4% vs. 23.1%) (*p* < 0.001). Similarly, most patients were admitted to urban teaching hospitals (72.0% vs. 70.8%), followed by urban non-teaching hospitals (20.0% vs. 19.7%) (*p* < 0.001), and to large hospitals (45.0% vs. 45.6%), followed by medium-sized hospitals (30.5% vs. 29.9%) (*p* = 0.15).

**Figure 2:**
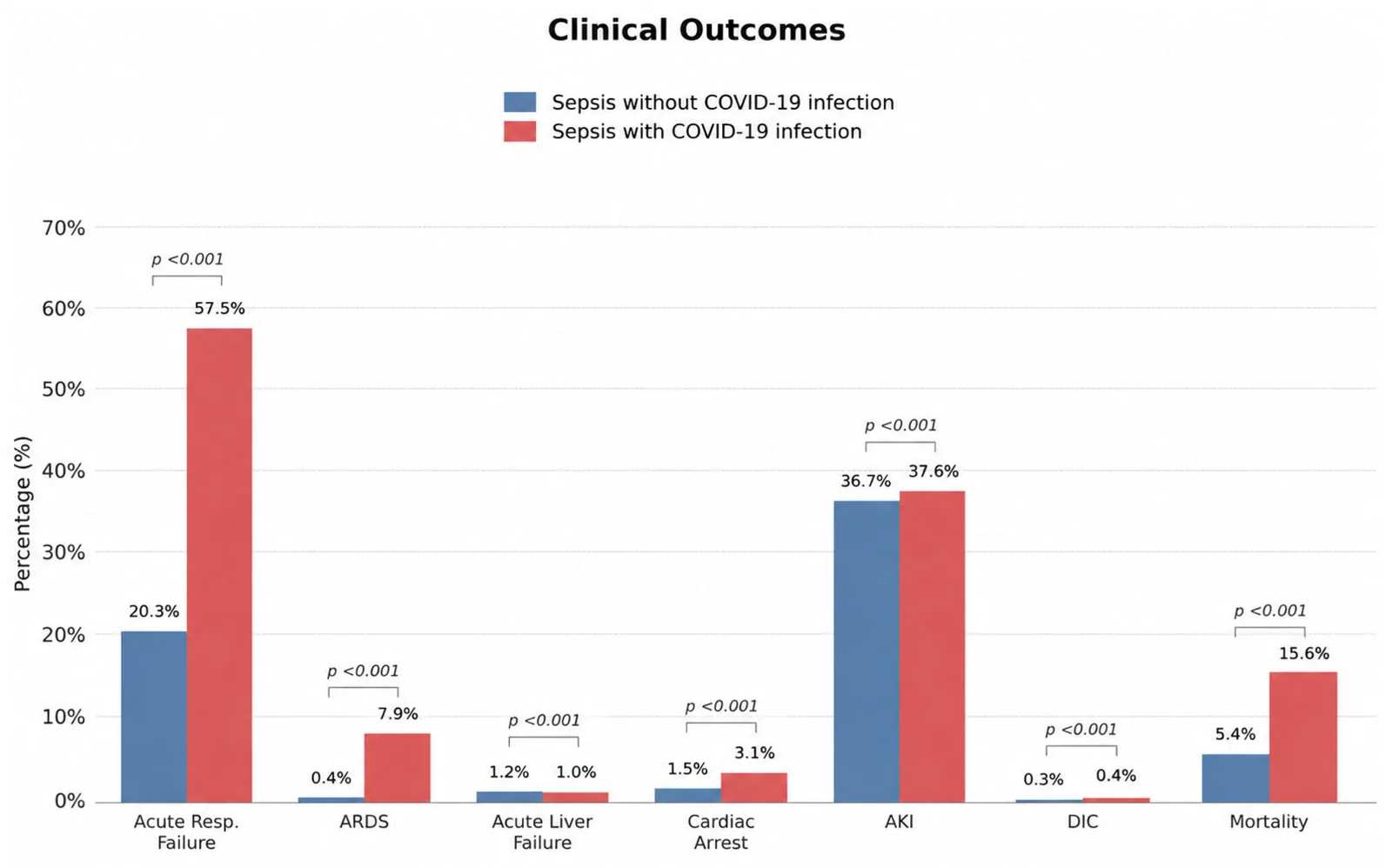
Clinical outcomes including acute respiratory failure, acute respiratory distress syndrome (ARDS), acute liver failure, cardiac arrest, acute kidney injury (AKI), disseminated intravascular coagulation (DIC), and in-hospital mortality among patients hospitalized with sepsis, with and without COVID-19 infection.

**Figure 3:**
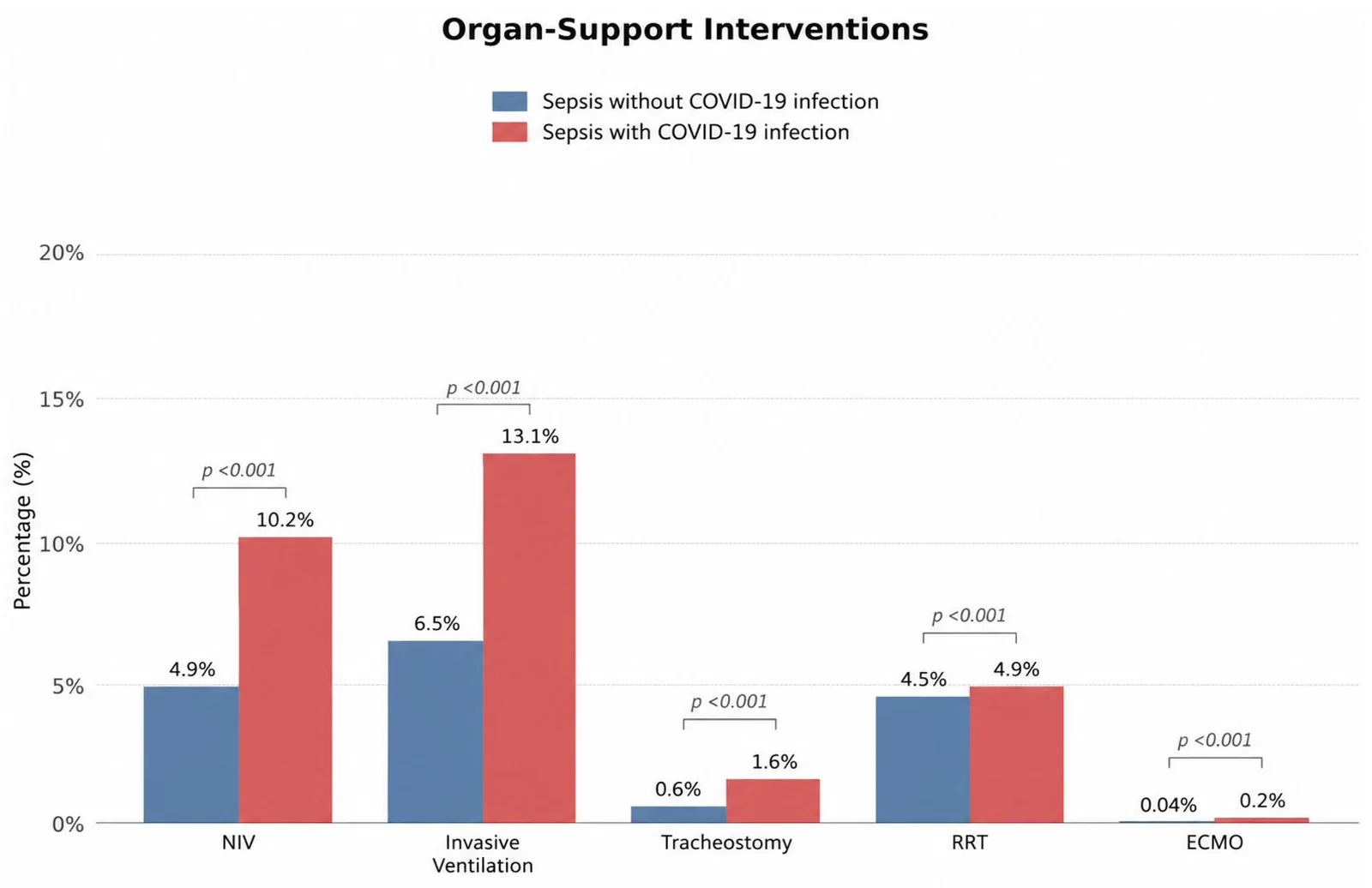
Organ-support interventions including noninvasive ventilation (NIV), invasive ventilation, tracheostomy, renal replacement therapy (RRT), and extracorporeal membrane oxygenation (ECMO) among patients hospitalized with sepsis, with and without COVID-19 infection.

Compared with patients with sepsis without COVID-19 infection, those with concomitant COVID-19 infection had significantly higher rates of clinical outcomes, including acute respiratory failure (57.5% vs. 20.3%; aOR 5.48; 95% CI 5.37–5.59), ARDS (7.9% vs. 0.4%; aOR 18.35; 95% CI 17.59–19.15), cardiac arrest (3.1% vs. 1.5%; aOR 2.19; 95% CI 2.11–2.27), acute kidney injury (37.6% vs. 36.7%; aOR 1.11; 95% CI 1.10–1.13), disseminated intravascular coagulation (0.4% vs. 0.3%; aOR 1.52; 95% CI 1.39–1.66), and in-hospital mortality (15.6% vs. 5.4%; aOR 3.70; 95% CI 3.62–3.79) (all *p* < 0.001) [Figure 2]. Organ-support interventions were also more frequent among patients with concomitant COVID-19 infection, including noninvasive ventilation (10.2% vs. 4.9%; aOR 2.34; 95% CI 2.29–2.40), invasive ventilation (13.1% vs. 6.5%; aOR 2.24; 95% CI 2.19–2.29), tracheostomy (1.6% vs. 0.6%; aOR 2.53; 95% CI 2.40–2.66), renal replacement therapy (4.9% vs. 4.5%; aOR 1.30; 95% CI 1.26–1.33), and extracorporeal membrane oxygenation (0.2% vs. 0.04%; aOR 4.29; 95% CI 3.72–4.96) (all *p* < 0.001) [Figure 3]. Acute liver failure was slightly less frequent among patients with sepsis and concomitant COVID-19 infection than among those without COVID-19 infection (1.0% vs. 1.2%; aOR 0.93; 95% CI 0.88–0.98; *p* = 0.006).

**Figure 4:**
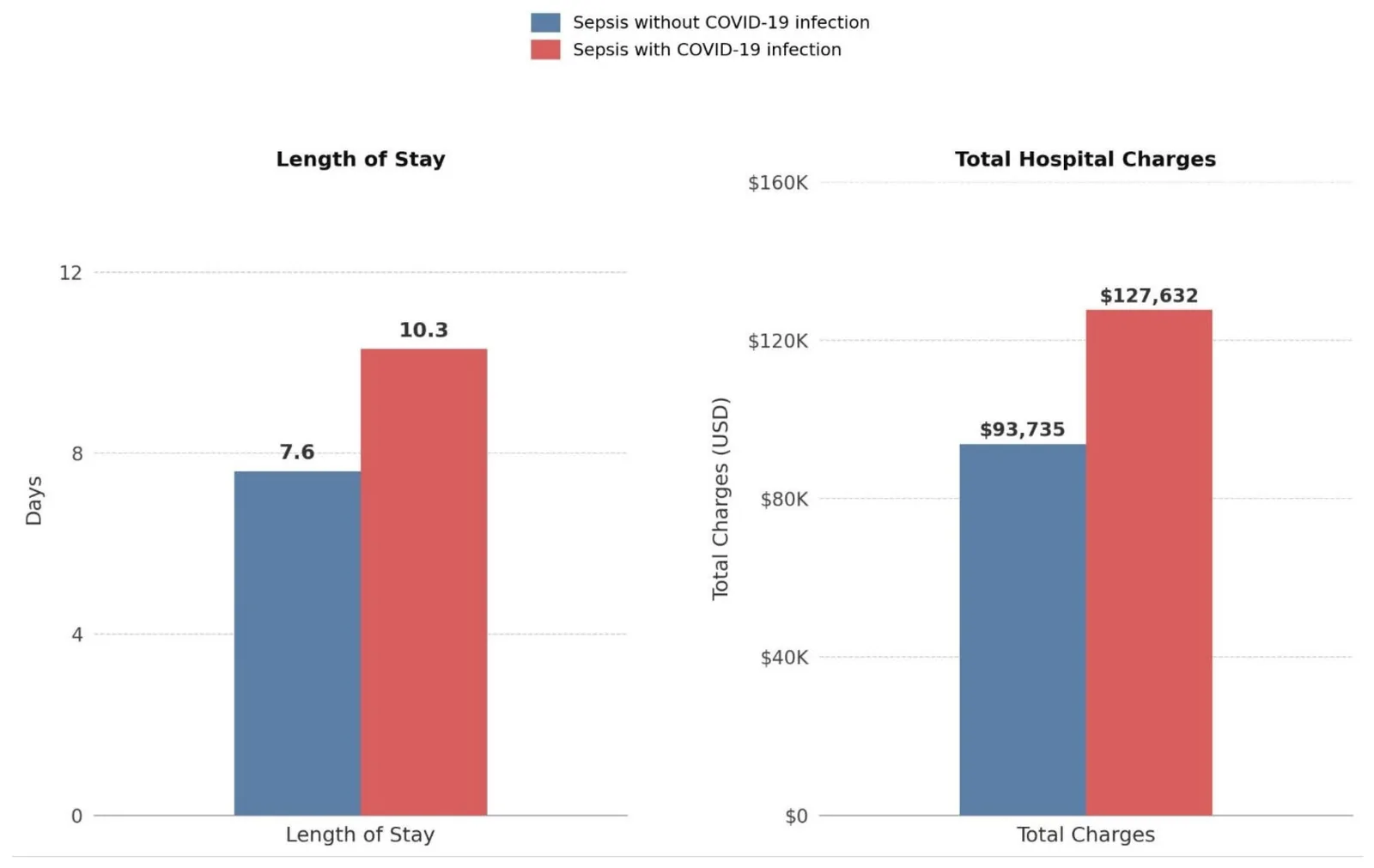
Length of hospital stay and Total hospital charges for patients hospitalized with sepsis, with and without COVID-19 infection.

**Figure 5:**
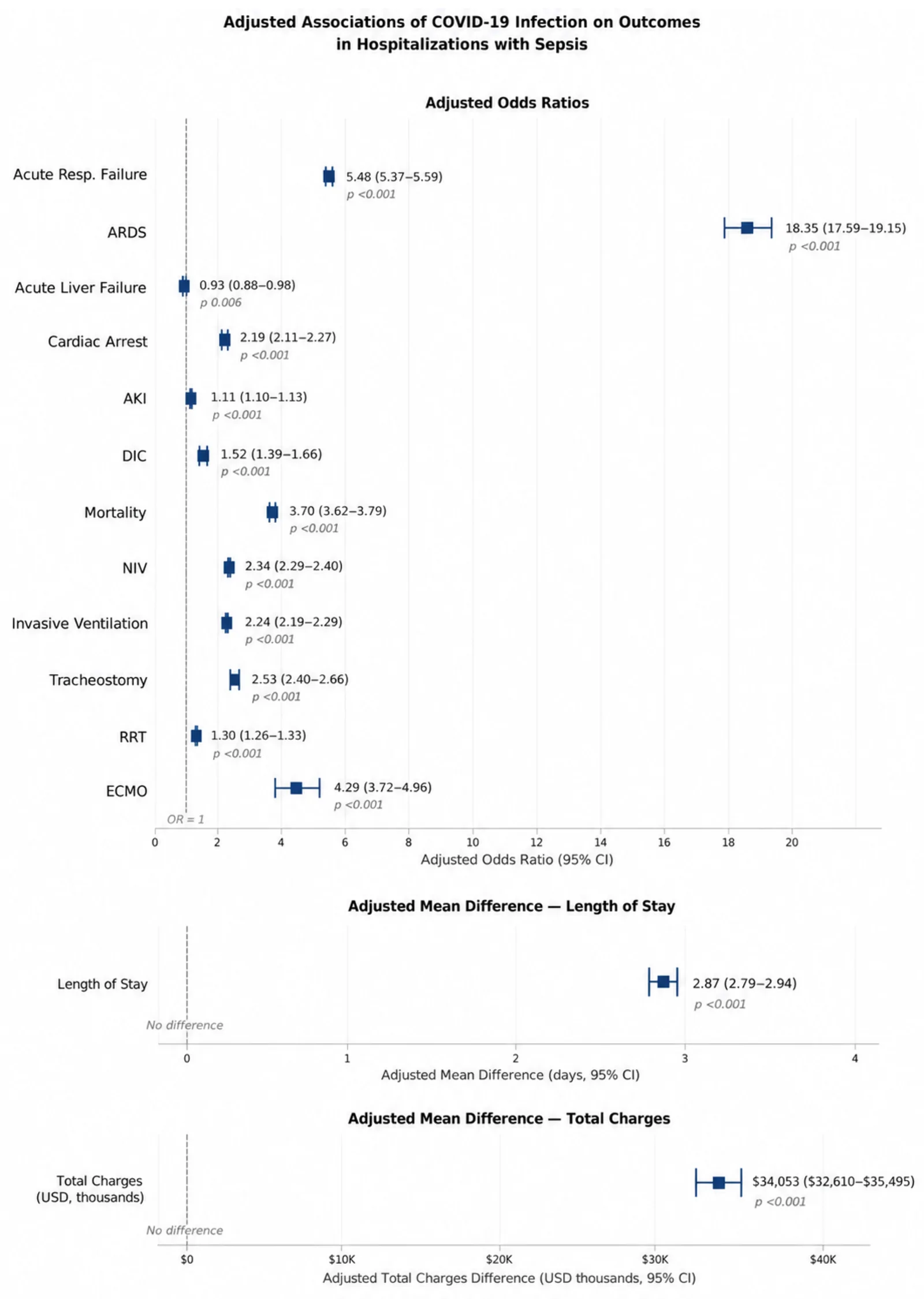
Forest plot depicting Adjusted Odds Ratios for Mortality, Invasive ventilation, Non-invasive ventilation, Acute kidney injury, Renal replacement therapy, Acute respiratory distress syndrome, Extracorporeal membrane oxygenation, cardiac arrest, Acute liver failure, Disseminated intravascular coagulation, Acute respiratory failure, Tracheostomy, and Adjusted Mean Differences for hospital length of stay and total hospital charges for patients with sepsis and concomitant COVID-19 infection.

Patients with concomitant COVID-19 infection also had significantly longer hospital stays (10.3 vs. 7.6 days; adjusted mean difference 2.87 days, 95% CI 2.79–2.94) and higher total hospitalization charges ($127,632 vs. $93,735; adjusted mean difference $34,053; 95% CI $32,610–$35,495) (both *p* < 0.001) [Figure 4 and Figure 5]. Sensitivity analyses using survey-weighted generalized linear models with a gamma distribution and log link yielded consistent findings for both length of stay and total hospital charges (Supplementary Table S3).

**Figure 6:**
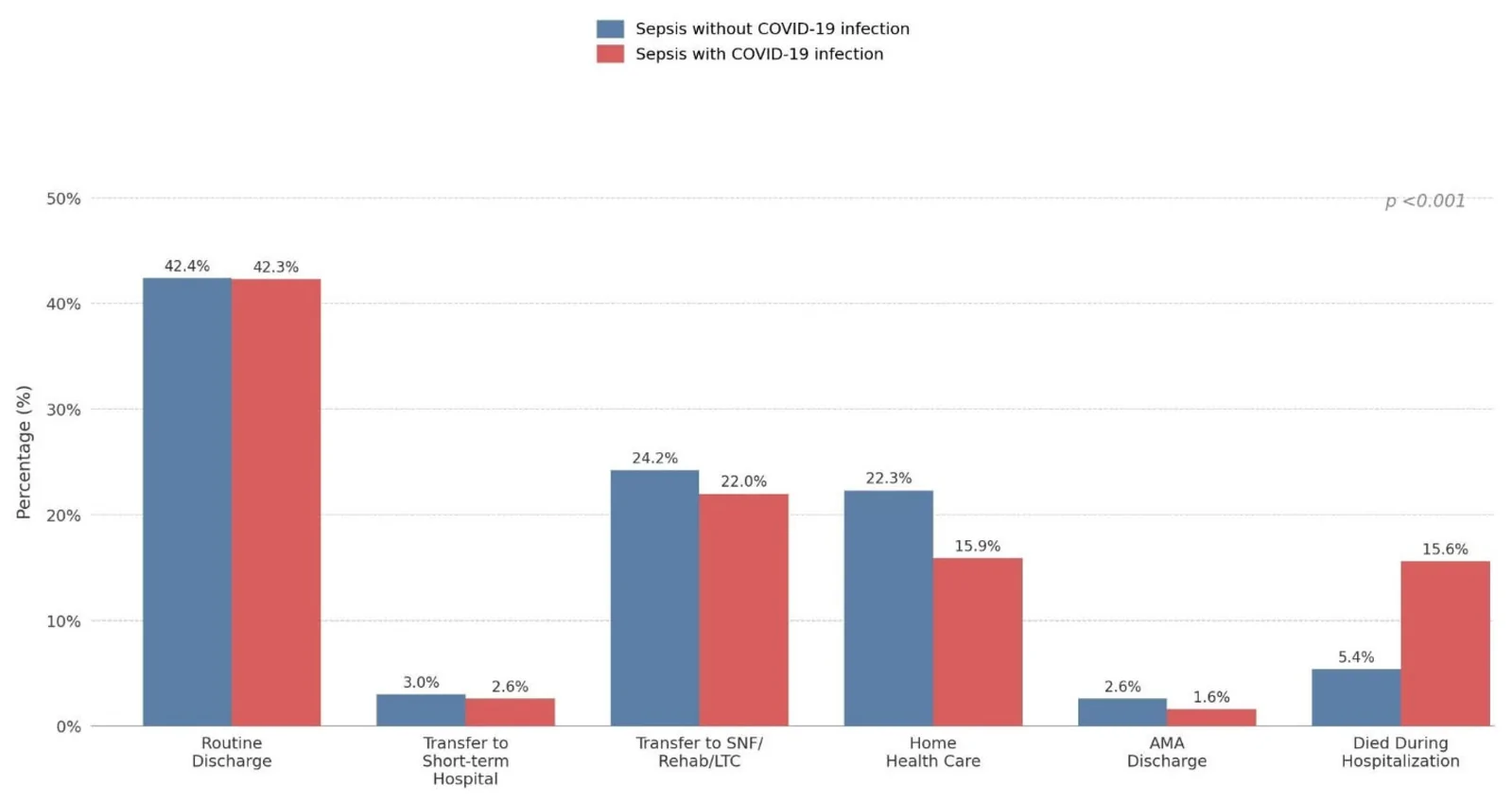
Discharge destinations for patients hospitalized with Sepsis, with and without COVID-19 infection.

Discharge destinations: Most of the patients from both cohorts had a routine discharge (42.3% vs. 42.4%), followed by discharge to a rehabilitation facility/long-term care/skilled nursing facility (SNF) (22.0% vs. 24.2%) (*p* <0.001) [Figure 6].

**Figure 7:**
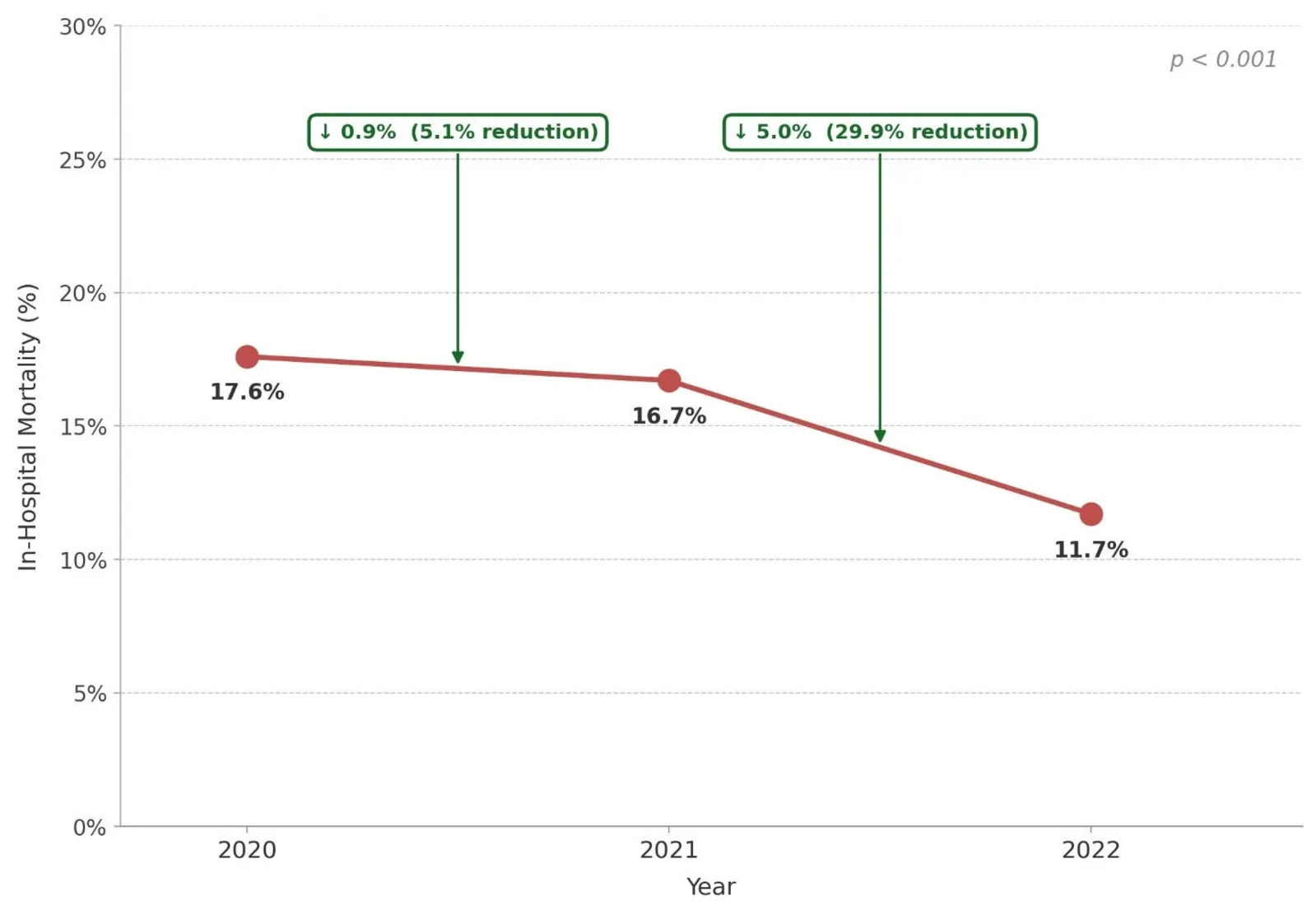
Mortality trend in patients with Sepsis and concomitant COVID-19 infection from the years 2020–2022.

**Figure 8:**
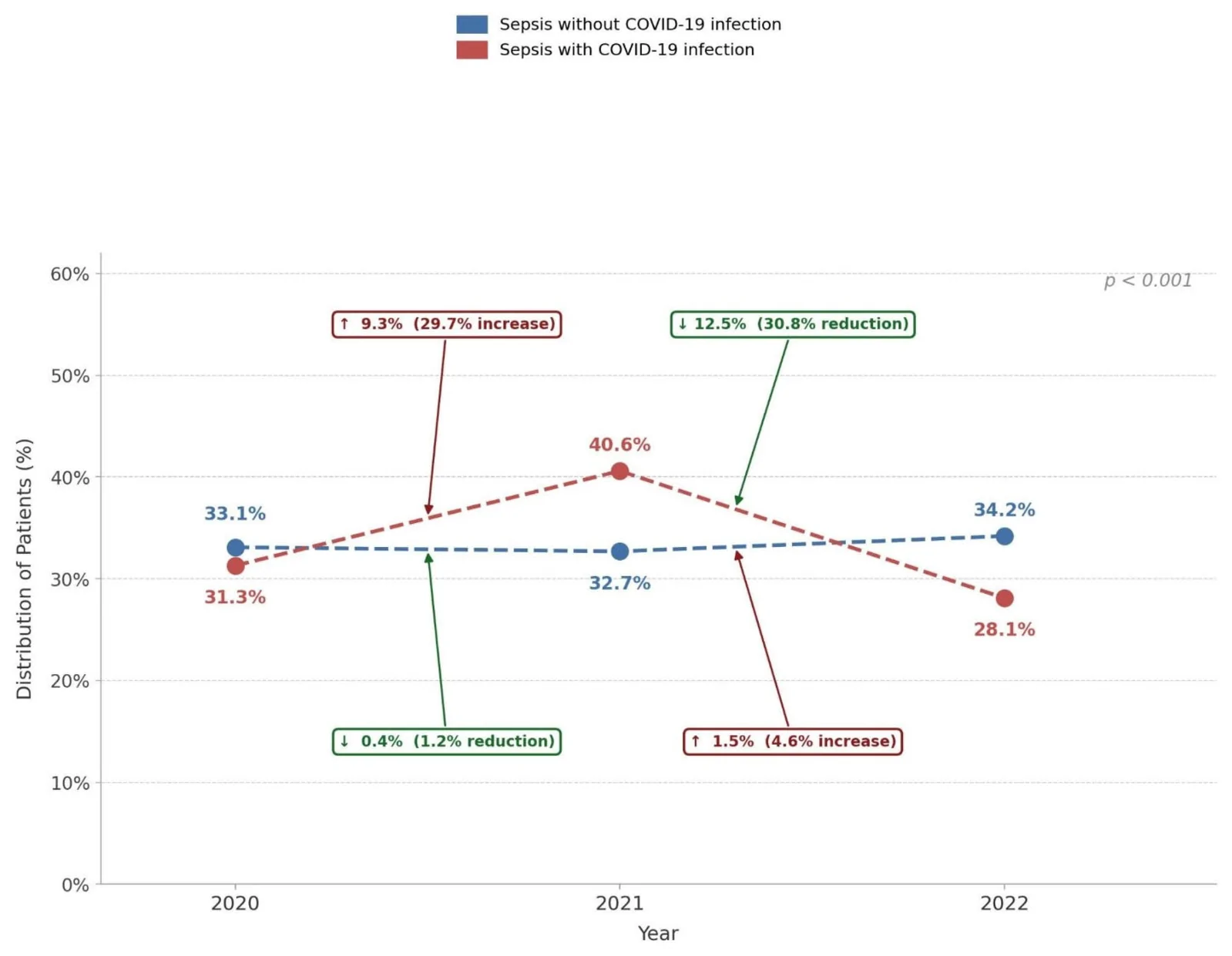
Distribution of patients with COVID-19 Infection and Sepsis in the years 2020–2022.

Temporal analysis demonstrated that in-hospital mortality among patients with sepsis with concomitant COVID-19 infection was the highest in 2020 (17.6%) and subsequently declined to 16.7% in 2021 and 11.7% in 2022 (*p* < 0.001) [Figure 7]. However, patients hospitalized with sepsis and concomitant COVID-19 were most frequent in 2021 (40.6%), whereas hospitalizations without COVID-19 infection were highest in 2022 (34.2%) (*p* < 0.001) [Figure 8].

## 4. Discussion

The COVID-19 pandemic revealed that sepsis associated with SARS-CoV-2 accounted for nearly 28.2% of COVID-19 hospitalizations and carried mortality rates exceeding 30% early in the pandemic [3]. This suggests that sepsis associated with COVID-19 has distinct clinical characteristics compared with bacterial sepsis. Sepsis is associated with substantial inpatient morbidity and mortality, even among patients without major pre-existing comorbidities [10]. In the United States, sepsis-related hospitalizations increased in the years preceding and during the COVID-19 pandemic. National HCUP data showed that sepsis-related inpatient stays increased from 1.8 million in 2016 to 2.1 million in 2019 and further increased to 2.5 million in 2021 after the emergence of COVID-19. Although in-hospital mortality declined before the pandemic, it increased during the COVID-19 pandemic [11]. The COVID-19 pandemic and its implications on sepsis-related outcomes and the use of hospital resources remain unclear and incompletely characterized in the literature. We analyzed the association between concurrent COVID-19 infection and in-hospital outcomes in adult patients hospitalized with sepsis, using the National Inpatient Sample (NIS) database for 2020–2022.

Patients with concomitant COVID-19 infection had more than eighteen-fold higher adjusted odds of ARDS representing one of the strongest associations observed in our analysis. These findings highlight the substantial burden of severe respiratory failure among patients hospitalized with sepsis and concurrent COVID-19 infection. This finding is consistent with the diffuse inflammatory lung injury characteristic of severe SARS-CoV-2 infection and likely explains the increased requirements for mechanical ventilation, ECMO, and prolonged intensive care. COVID-19 preferentially causes severe acute hypoxemic respiratory failure due to diffuse alveolar damage, pneumonitis, and progression to ARDS, which may be especially dramatic given sepsis-related immune suppression [12,13,14].

The incidence of acute kidney injury was slightly higher among patients with sepsis with concurrent COVID-19 infection than among those without COVID-19. Concurrent SARS-CoV-2 infection may further contribute to renal injury in patients with sepsis and increase the likelihood of severe kidney injury requiring dialysis [15]. Although disseminated intravascular coagulation was relatively uncommon, its increased adjusted odds are consistent with the profound endothelial activation and thrombo-inflammatory state characteristic of severe COVID-19 [16]. These findings support previous reports demonstrating persistent activation of coagulation pathways in SARS-CoV-2 infection. The predominance of respiratory complications is consistent with an APACHE IV-based cohort study, which found that respiratory sepsis was associated with the highest requirement for invasive ventilation and the longest ICU stay among the evaluated sources of sepsis [17]. Taken together, these findings suggest that the pulmonary origin of infection may contribute importantly to the clinical severity and resource requirements of sepsis with concurrent COVID-19 infection [17]. Cardiac arrest occurred more than twice as often among patients with sepsis with concurrent COVID-19 infection. Potential mechanisms include profound hypoxemia, systemic inflammation, myocardial injury, arrhythmia, and cardiac dysfunction [18]. The lower adjusted risk of acute liver failure may reflect the predominance of respiratory rather than hepatic organ failure in sepsis with concurrent COVID-19 infection.

ECMO utilization was also substantially higher among patients with sepsis and concomitant COVID-19 infection, with more than four-fold higher adjusted odds of ECMO support compared with patients with sepsis without COVID-19 infection. Invasive mechanical ventilation was significantly higher in the COVID-19 sepsis group. In contrast, NIV utilization in the COVID-19 cohort was notably higher, as is consistent with the burden of respiratory compromise seen in these populations and the efforts to avoid invasive mechanical ventilation whenever feasible [19]. Similarly, the need for renal replacement therapy was greater in the COVID-19 cohort. The combined need for ventilatory support in both cases had immense implications for resource planning, putting tremendous pressure on critical care infrastructure as the pandemic surged to the peak. These findings are consistent and extend our preliminary analysis, which demonstrated increased mortality, invasive and non-invasive mechanical ventilation with patients with concomitant sepsis and COVID-19 [20,21]. The higher rate of tracheostomy likely reflects prolonged mechanical ventilation among patients with COVID-19-associated respiratory failure.

Our results indicate that adults presenting with sepsis with concurrent COVID-19 infection had significantly worse mortality, mechanical ventilation (noninvasive and invasive), and length of stay. Patients with sepsis who had COVID-19 showed higher in-hospital mortality compared with patients who did not have COVID-19. These results are in line with early pandemic-associated reports describing high case fatality rates in acutely ill COVID-19 patients [9,22]. A potential explanation for these findings may be that SARS-CoV-2 induces a hyperinflammatory state, including cytokine storm, aberrant innate immune responses, endothelial injury, and coagulopathy, that amplify the pathophysiologic cascade of sepsis [16,23]. When this hyperinflammatory state is superimposed on a background of chronic medical conditions as well as immune dysregulation secondary to sepsis, there is an accelerated multiorgan failure and death [24].

Temporal trends in mortality among these patients were also examined and showed a significant decline from 17.6% in 2020 to 11.7% in 2022. Following the same trend, COVID–sepsis hospitalizations peaked in 2021. The higher burden observed in 2021 may partially reflect the predominance of the Delta variant, which has been associated with increased disease severity and hospitalization compared with earlier strains [25,26]. The follow-up decline in 2022 is probably multifactorial: development and access to COVID-19 vaccines, lower-grade Omicron variant, increased therapeutic efficacy, and accumulated provider experience in the treatment of critical illness caused by COVID-19 infection [27,28,29]. This temporal improvement is consistent with other NIS-based analyses showing improved outcomes among COVID-19-associated hospitalizations across the later pandemic period [30,31,32].

Hospital length of stay in sepsis patients with COVID-19 was significantly prolonged. The longer hospital stay likely reflects greater illness severity, higher rates of respiratory failure, prolonged ventilatory support, and multiorgan dysfunction among patients with concurrent COVID-19 infection [33]. Prolonged hospitalization is associated with increased healthcare resource utilization and costs, reflecting the greater severity of illness and complexity of care required for critically ill patients [34]. Our study demonstrated that patients with sepsis and concurrent COVID-19 incurred hospital charges that were higher after adjustment, likely reflecting greater disease severity, associated complications, and the need for intensive multidisciplinary care. These findings underscore the substantial economic burden of sepsis with concurrent COVID-19 infection on the U.S. healthcare system. Although discharge disposition was broadly similar between groups, differences likely reflect the higher in-hospital mortality and prolonged hospitalization among patients with sepsis with concurrent COVID-19 infection.

Demographically, patients affected by sepsis and COVID-19 were of the same mean age and more likely to be male. These results are consistent with well-established evidence indicating that male sex and older age represent independent risk factors for severe COVID-19, potentially related to sex-associated variations in immune regulation and increased rates of comorbidity in older individuals [35,36]. Racial and ethnic disparities were also found, with Hispanic patients constituting a higher portion of the total COVID-19 sepsis group and African Americans being slightly overrepresented, while Caucasian patients were underrepresented. These discrepancies are likely due to the well-known disparity in the burden of COVID-19 among racialized and ethnic minority populations, which also are influenced by social determinants of health such as occupational exposure, housing density, access to health services, and high baseline prevalence of comorbid conditions [37,38]. Insurance status across groups was largely similar, with Medicare being the primary payer accounting for more than 50% in both cohorts. On the other hand, a slightly higher proportion of the patients in the COVID-19 sepsis group had private insurance and the uninsured rate was slightly lower. The nuanced differences here may be due to pandemic changes in insurance coverage, as well as the demographics of populations most affected by serious COVID-19 during the study time. Notably, the lowest income quartile patients represented nearly a third of both groups, again confirming the socioeconomic vulnerability of patients hospitalized with sepsis irrespective of COVID-19. Although patients with concurrent COVID-19 had a lower proportion of hospitalizations with a Charlson Comorbidity Index ≥3, COVID-19 remained independently associated with substantially worse outcomes after multivariable adjustment, suggesting that the observed association of SARS-CoV-2 extended beyond baseline comorbidity burden. This is biologically plausible because SARS-CoV-2 can cause viral sepsis with systemic inflammation, endothelial injury, coagulopathy, and multiorgan dysfunction [24]. After adjustment for these variables, both mortality and resource intensity remained significantly high in the COVID-19 sepsis group, indicating that concurrent COVID-19 infection remained independently associated with adverse sepsis outcomes beyond the extent of conventional comorbidity indices. Hospital characteristics were largely similar from group to group. Urban teaching hospitals represented the majority of admissions of more than 70% in both cohorts, whereas large-bed hospitals accounted for roughly half of admissions in both cohorts. This mirrors the higher rate of severe sepsis and COVID-19 cases at tertiary and academic centers, which are ideal to handle complex, critical patients, but are more susceptible to capacity constraints when there are heavy pandemic surges [39].

## 5. Strengths

Our study is noted for a number of significant strengths. Using the NIS as the country’s largest all-payer inpatient database affords us a nationally representative sample with strong statistical power and generalizability. The large sample sizes (5,933,442 sepsis subjects without COVID-19 and 939,864 with COVID-19) enable rigorous adjustment with multiple factors and accurate estimates of odds ratios with small confidence intervals. Our study covers three (3) years (2020–2022), allowing us to see the dynamic status of the pandemic at different stages of its trajectory and providing access to temporal trend analysis, which increases the robust and interpretative value of our outcomes. Unlike earlier studies restricted to single health systems or the first pandemic wave, the present analysis captures evolving outcomes during successive SARS-CoV-2 variants and changes in clinical management.

## 6. Limitations

There are a few limitations in this study to be acknowledged. As an administrative claims database, the NIS is based on ICD-10-CM diagnostic and procedural codes; therefore, coding variability and misclassification of sepsis or COVID-19 are possible. Because COVID-19 infection was identified using ICD-10-CM code U07.1 during the same hospitalization, this study confirms concurrent COVID-19 infection among patients with sepsis but cannot establish SARS-CoV-2 as the causal etiology of sepsis. The NIS does not allow for further analysis of specific clinical information, such as severity-of-illness scores, microbiological testing, treatments performed, vaccination status, infecting SARS-CoV-2 variant, or causes of death, restricting the breadth of interpretation of the etiologic processes. The distribution of sepsis code categories differed significantly between cohorts, with A40 streptococcal sepsis less frequent among hospitalizations with concurrent COVID-19 infection and A41 other sepsis more frequent. These differences should be interpreted cautiously because administrative coding does not allow definitive determination of the microbiologic etiology of sepsis. In addition, some COVID-19-related viral sepsis encounters may have been coded within A41 categories, including A41.89 (other specified sepsis) or A41.9 (sepsis, unspecified organism), which may have contributed to the observed differences in sepsis-code distribution between groups and to residual confounding or coding-related bias. To address this concern, we performed stratified subgroup analyses by A40 and A41 code category, which are presented in Supplementary Table S2. Furthermore, although we accounted for relevant factors such as age, sex, race, insurance status, comorbidity burden, hospital region, and teaching status, residual confounding from unmeasured factors cannot be excluded. Finally, the NIS captures inpatient encounters only and does not include post-discharge mortality or long-term outcomes, which may underestimate the overall burden of mortality associated with sepsis and concurrent COVID-19 infection.

## 7. Future Directions

Further prospective multicenter studies are needed to better characterize the association between concurrent COVID-19 infection and outcomes among patients with sepsis. Collecting detailed clinical data, vaccination status, viral variants, microbiologic findings, severity of illness, timing of antiviral and antimicrobial therapy, corticosteroid use, and long-term outcomes would provide a clearer understanding of the factors driving worse outcomes and help inform future treatment strategies.

## 8. Conclusions

In this large, nationally representative study of hospitalized adults with sepsis in the United States over the period 2020 through 2022, concurrent COVID-19 infection was independently associated with much higher in-hospital mortality, showed a higher requirement for invasive and non-invasive mechanical ventilation, and significantly increased length of stay. These findings underscore the importance of early recognition and aggressive management of patients with sepsis and concurrent COVID-19 infection. The decline in mortality observed over the study period should be interpreted in the context of broader changes in the pandemic, including evolving SARS-CoV-2 variants, increasing vaccine availability, advances in COVID-19 therapeutics, and accumulated clinical experience in the management of severe COVID-19 infection [40,41]. More prospective studies using clinical severity scores, vaccination status, and variant-specific data are needed to characterize the mechanistic interplay between COVID-19 and sepsis further and address modifiable targets for intervention.

## Figures and Tables

**Table 1:** Demographic and Clinical Characteristics of Hospitalized Patients with Sepsis, stratified by COVID-19 infection status.

Variable	Overall Adult Sepsis Cohort (*N* = 6,873,306)	Sepsis Without COVID-19 Infection (*N* = 5,933,442)	Sepsis with COVID-19 Infection (*N* = 939,864)	*p*-Value
Age, mean (SD), years	63.8 (17.6)	63.8 (17.7)	63.8 (17.2)	0.82
Sex				<0.001
Male	52.3%	51.8%	55.6%	
Female	47.7%	48.2%	44.4%	
Race				<0.001
Caucasian	65.6%	67.2%	55.8%	
African American	14.8%	14.3%	17.6%	
Hispanic	12.8%	11.7%	18.5%	
Asian/Pacific Islander	3.1%	3.0%	3.5%	
Native American	0.8%	0.8%	0.7%	
Other	2.9%	2.7%	3.7%	
National income quartile				<0.001
1–$38,999	31.1%	31.0%	31.7%	
$39,000–$47,999	26.2%	26.2%	26.3%	
$48,000–$62,999	23.4%	23.4%	23.9%	
>$63,000	19.2%	19.4%	18.1%	
Insurance				<0.001
Medicare	58.9%	59.4%	55.2%	
Medicaid	16.5%	16.8%	14.8%	
Private	20.4%	19.5%	26.0%	
Uninsured	4.3%	4.3%	4.0%	
Charlson Comorbidity Index				<0.001
0	19.0%	18.3%	23.3%	
1	19.6%	18.9%	24.1%	
2	17.8%	17.9%	17.0%	
≥3	43.7%	44.9%	35.6%	
Hospital region				<0.001
Northeast	15.9%	15.7%	16.6%	
Midwest	19.4%	19.7%	17.7%	
South	41.6%	41.4%	43.2%	
West	23.1%	23.1%	22.4%	
Hospital location and teaching status				<0.001
Rural	9.2%	9.4%	7.9%	
Urban, non-teaching	19.8%	19.7%	20.0%	
Urban, teaching	71.0%	70.8%	72.0%	
Hospital bed size				0.15
Small	24.4%	24.4%	24.4%	
Medium	30.0%	29.9%	30.5%	
Large	45.6%	45.6%	45.0%	
A40 streptococcal sepsis	2.8%	3.1%	1.0%	<0.001
A41 other sepsis	97.1%	96.8%	98.9%	<0.001

NIS = National Inpatient Sample; COVID-19 = Coronavirus Disease 2019.

## Data Availability

The data presented in this study are openly available in Healthcare Cost and Utilization Project (HCUP) Central Distributor at https://hcup-us.ahrq.gov/nisoverview.jsp (accessed on 15 March 2026), reference number: HCUP- 28M71GVP5.

## Supplementary Materials

The following supporting information can be downloaded at: https://www.mdpi.com/article/10.3390/idr18050118/s1, Table S1: Sepsis ICD-10-CM Code Composition by Concurrent COVID-19 Status, Table S2: Adjusted Outcome Models Stratified by A40 and A41 Sepsis Code Category, Table S3: Distributional Diagnostics and Gamma-Log Sensitivity Analyses for Length of Stay and Total Charges.
