# Supplementary material for: Clinical Outcomes and Hospital Resource Utilization Among Patients with Sepsis and COVID-19 Infection: Insights from the United States National Inpatient Sample Analysis, 2020–2022
Source: Infect Dis Rep. 2026 Oct 10;18(5):118. doi: 10.3390/idr18050118 (PMC13704002; doi:10.3390/idr18050118)
Supplement: Supplementary file 1 [file idr-18-00118-s001.zip › idr-4518080-supplementary.pdf]

infectious disease reports

MDPI

Supplementary Table S1. Sepsis ICD-10-CM Code Composition by Concurrent COVID-19 Status.

Code category	Definition	Overall cohort	Sepsis without COVID-19	Sepsis with COVID-19	P-value
A40	Streptococcal sepsis	2.8%	3.1%	1.0%	<0.001
A41	Other sepsis	97.1%	96.8%	98.9%	<0.001
A41.89	Other specified sepsis	12.8%	3.4%	72.1%	<0.001
A41.9	Sepsis, unspecified organism	65.3%	72.4%	20.8%	<0.001
R65.20	Severe sepsis without septic shock	24.4%	24.0%	27.0%	<0.001

Supplementary Table S2. Adjusted Outcome Models Stratified by A40 and A41 Sepsis Code Category.

Outcome	A40 aOR	A40 95% CI	A40 P-value	A41 aOR	A41 95% CI	A41 P-value
Mortality	4.95	4.14-5.92	<0.001	3.67	3.58-3.75	<0.001
Acute respiratory failure	2.64	2.36-2.94	<0.001	5.52	5.41-5.63	<0.001
ARDS	18.61	13.91-24.89	<0.001	18.30	17.53-19.10	<0.001
Non-invasive ventilation	1.74	1.44-2.11	<0.001	2.35	2.29-2.41	<0.001
Invasive ventilation	2.93	2.50-3.43	<0.001	2.23	2.17-2.28	<0.001
ECMO	13.39	4.39-40.88	<0.001	4.22	3.65-4.88	<0.001
AKI	1.34	1.20-1.49	<0.001	1.11	1.10-1.13	<0.001
Renal replacement therapy	1.52	1.21-1.91	<0.001	1.29	1.26-1.33	<0.001
Cardiac arrest	3.49	2.46-4.95	<0.001	2.16	2.09-2.24	<0.001

DIC	2.07	1.07-4.00	0.030	1.51	1.38-1.65	<0.001
Acute liver failure	1.02	0.62-1.70	0.931	0.93	0.88-0.98	0.005
Tracheostomy	3.73	2.50-5.56	<0.001	2.51	2.38-2.64	<0.001

Supplementary Table S3. Distributional Diagnostics and Gamma-Log Sensitivity Analyses for Length of Stay and Total Charges.

Outcome	Group	Mean	Median	SD	Skewness	Kurtosis	Adjusted linear result	Gamma-log GLM ratio	P-value
Length of stay	Overall adult sepsis	7.94	5	9.88	7.59	130.93			
Length of stay	Sepsis without COVID-19	7.57	5	9.49	8.15	149.68			
Length of stay	Sepsis with COVID-19	10.29	7	11.82	5.63	74.47	2.87 days (2.79-2.94)	1.42 (1.41-1.43)	<0.001
Total charges	Overall adult sepsis	$98,371	$54,667	$169,892	12.03	328.86			
Total charges	Sepsis without COVID-19	$93,735	$52,581	$162,160	12.53	356.32			
Total charges	Sepsis with COVID-19	$127,632	$70,020	$209,946	9.98	228.18	$34,053 (32,610-35,495)	1.41 (1.39-1.42)	<0.001

https://doi.org/10.3390/idr18050118
